# Evolutionary analysis of seasonal influenza A viruses in Pakistan 2020–2023

**DOI:** 10.1111/irv.13262

**Published:** 2024-02-22

**Authors:** Nazish Badar, Aamer Ikram, Muhammad Salman, Sidra Saeed, Hamza Ahmed Mirza, Abdul Ahad, Massab Umair, Umer Farooq

**Affiliations:** ^1^ Public Health Laboratories Division National Institute of Health Islamabad Pakistan; ^2^ National Institute of Health Islamabad Pakistan; ^3^ National Agricultural Research Center Islamabad Pakistan

**Keywords:** influenza A, Pakistan, sequence analysis

## Abstract

**Introduction:**

Influenza A viruses cause global health concerns due to their high amino acid substitution rates. They are linked to yearly seasonal epidemics and occasional pandemics. This study focused on sequencing influenza A virus strains in Pakistan.

**Materials and Methods:**

We analyzed the genetic characteristics of influenza A(H1N1)pdm09 and A(H3N2) viruses circulating in Pakistan from January 2020 to January 2023. Whole genome sequences from influenza A (*n* = 126) virus isolates were amplified and sequenced by the Oxford Nanopore (MinION) platform.

**Results:**

The HA genes of influenza A(H1N1)pdm09 underwent amino acid substitutions at positions K54Q, A186T, Q189E, E224A, R259K, and K308R in sequenced samples. The HA genes of influenza A(H3N2) had amino acid substitutions at G53D, E83K, D104G, I140M, S205F, A212T, and K276R in the sequenced samples. Furthermore, the HA gene sequences of influenza A(H1N1)pdm09 in this study belonged to subclade 6B.1A.5a.2a. Similarly, the HA gene sequences of influenza A(H3N2) were classified under six subclades (3C.3a.1 and 3C.2a1b.2a [2, 2a.1, 2b, 2c, and 2a.3b]). Notably, amino acid substitutions in other gene segments of influenza A(H1N1)pdm09 and A(H3N2) were also found.

**Conclusion:**

These findings indicate influenza A(H1N1)pdm09 and A(H3N2) viruses co‐circulated during the 2020–2023 influenza season in Pakistan. Continued surveillance is crucial for real‐time monitoring of possible high‐virulence variation and their relevance to existing vaccine strains.

## 1 INTRODUCTION

The influenza A virus (IAV) (Family: *Orthomyxoviridae*, Genus: *Alphainfluenzavirus*) is a single‐stranded RNA virus of negative sense that infects a wide range of hosts, including aquatic birds, humans, and pigs.^1^ The 13.5‐kb influenza A virus genome has eight RNA segments. These segments, namely, RNA segments 1, 2, 4, 5, and 6, encode polymerase‐basic 2 (PB2), polymerase‐acidic (PA), hemagglutinin (HA), nucleoprotein (NP), and neuraminidase (NA) viral proteins, respectively. The remaining segments encode more than one viral protein. Notably, HA, NA, and matrix protein 2 (M2) ion channel protein are positioned on the virus surface and are susceptible to immune targeting. In contrast, M1 underlies these membrane proteins and forms the core of the virus. Polymerase‐basic 1 (PB1), PB2, PA, NP, matrix protein 1 (M1), non‐structural protein (NS1), and nuclear export protein (NEP) are situated within the lipid envelope. The influenza virus is a significant health concern worldwide due to its annual seasonal epidemics and occasional pandemics, resulting in high morbidity and mortality rates in the population.^2^, ^3^ New viral variants appear regularly due to viral genetic and antigenic variation caused by mutation events such as nucleotide misincorporation during genome replication or the exchange of genomic segments.^4^ The impact of mutations on viral fitness, as well as host immunity‐related factors or ecological and environmental mechanisms, all influence the duration and recurrence of the emergence of new epidemic threats.^5^ As a result, active molecular‐based surveillance aimed at identifying patterns of viral evolution is a top priority in national policies tackling influenza disease prevention, regulation, and treatment.

To perform virus genetic characterization, public health laboratories have mainly depended on Sanger sequencing of the HA gene, which partly covers one of the virus's eight negative‐sense single‐stranded RNA segments.^4^ Furthermore, this method almost entirely focuses on the consensus sequences representing the dominant virus lineage within each infected host at a given time, limiting our understanding of intra‐patient virus population diversity and transmission dynamics.^6^, ^7^ With the increased availability of next‐generation sequencing (NGS) technologies that enable rapid and affordable whole‐genome sequencing (WGS), a new era of influenza surveillance based on genetic analysis of influenza viruses at the whole‐genome scale has begun.^8^, ^9^ The advancement of WGS using first‐ and second‐generation sequencers has greatly improved our understanding of IAV. However, challenges like high costs, long processing times, complex protocols, and bulky equipment have prompted the exploration of better methods. Third‐generation sequencers, especially the portable MinION nanopore device from Oxford Nanopore Technologies (ONT), have emerged as a promising solution. The MinION offers real‐time sequencing and potential multiplex barcoding capabilities. This approach has the potential to significantly reduce costs and time in WGS during outbreaks, thereby accelerating response efforts and aiding disease management.^10^, ^11^ The rapid evolution of the seasonal influenza virus genome sequence presents an underlying dilemma for vaccine development and selection. As a result, the current study aims to elucidate the molecular mechanisms of origin and genetic variability of influenza viruses in Pakistan from 2020 to 2023 and to monitor for the extent of mutation in our region, which is especially important for seasonal influenza prevention and control.

## 2 MATERIALS AND METHODS

### 2.1 Case definition for sample collection

Both outpatient and inpatient individuals were screened using the criteria recommended by the World Health Organization (WHO) to identify cases of influenza‐like illness (ILI) and severe acute respiratory infection (SARI). ILI cases were identified as individuals who developed a fever (>38°C) and cough or sore throat within 7 days of the onset of symptoms. SARI cases were defined as individuals who experienced a sudden onset of fever (>38°C), cough, or sore throat and required hospitalization within 7 days.

### 2.2 Study population

The National Influenza Center (NIC) in Islamabad Pakistan partnered with all provinces in the country to carry out efforts related to influenza surveillance. The Federal Government Services Hospital in Islamabad was the main sentinel location for sample collection. Other provincial sentinel sites included Hayatabad Medical Complex Hospital in Khyber Pakhtunkhwa, King Edward Medical University Hospital in Punjab, Civil Hospital in Sindh, and Bolan Medical Complex Hospital in Baluchistan. These hospitals were classified as influenza surveillance subcenters. Each week, a minimum of 5–10 throat and/or nasopharyngeal swabs samples were collected from suspected cases who met the inclusion criteria (case definition for ILI and SARI), as well as from case contacts and patients with a history of influenza.

### 2.3 Sample collection and RNA extraction

The samples were then transported in a universal transport medium (UTM, Copan Diagnostics) in a cold chain to the NIC, National Institutes of Health (NIH) Pakistan, and tested for IVA at the virology department. Viral RNA was isolated from the samples using MagMAX™ Viral/Pathogen Nucleic Acid Isolation Kit and KingFisher™ Flex Purification System (ThermoFisher Scientific, USA). The influenza Multiplex Assay Primers and Probes Kit by CDC (ThermoFisher Scientific, Waltham, USA) was used to identify the presence of influenza A virus in samples.^12^ Then influenza A subtyping primers of CDC were used to identify influenza A further in A(H1N1)pdm09 and A(H3N2) by using the TaqPathTM Real‐time RT‐PCR kit (ThermoFisher Scientific, Waltham, USA). Representative samples (*n* = 129) from different geographical regions collected at various time periods over 3 years were processed for sequencing (supporting information). The sequencing samples with Ct values <30 from years 2020 (*n* = 4), 2021 (*n* = 21), 2022 (*n* = 51), and 2023 (*n* = 53) were selected for Nanopore sequencing. Due to a failed QC following the cDNA enrichment phase, three samples were not processed further. All 126 study participants belonged to Islamabad, Karachi, Multan, Gilgit, and Muzaffarabad. The selected subjects ranged in age groups from 1–57 years old, with a median age of 29. The male‐to‐female ratio was 3:4.

### 2.4 Library preparation for Nanopore sequencing technology

Each purified RNA sample (3 μL) was used for multiplex RT‐PCR reactions. In this scheme, viral RNA segments of influenza A were simultaneously amplified using primers Uni12/Inf‐1, Uni12/Inf‐3, and Uni13 (Table 1) targeting the highly conserved sequence of viral RNA termini that are present at the ends of each genome segment.^13^ Multiplex RT‐PCR amplicons were generated by the SuperScript III One‐Step RT‐PCR System with Platinum Taq DNA Polymerase (ThermoFisher).

**TABLE 1:** Influenza A virus primers.

Component	Sequence	
Uni 12	ACGCGTGATCAGCAAAAGCAGG	Zhou et al.^13^
Uni 12	ACGCGTGATCAGCGAAAGCAGG	
Uni 13	ACGCGTGATCAGTAGAAACAAGG	

AMPure XP beads were used for the Purification of the PCR product. The ligation sequencing kit from ONT was used to prepare the libraries (SQK‐LSK109). Native barcoding expansion kits were used for multiplexing (EXP‐NBD‐104‐114). DNA repair and end‐preparation were carried out using the NEBNext Ultra II End‐Repair/dA‐tailing kit from New England Biolabs. To barcode the samples, 200 fmol of initial cDNA was combined with the native barcodes and the Blunt/TA Ligase Master Mix from New England Biolabs, and the reaction mixture was incubated at room temperature (15–25°C) for 20 min, followed by a 10‐min incubation at 65°C. For equal representation in the final library combination, equal numbers of indexed products were blended to increase the efficiency of barcode ligation by using smaller pieces. On each MinION flowcell, up to 12 clinical samples (90 fmol/sample) were multiplexed, with a no‐template control (NTC) processed in each pooled library phase. A DNA library was loaded onto a MinION Spot On flowcell that had been primed (R9.4). Samples were sequenced for 48 h using FLO‐MIN106 flowcells on MinION MK1b sequencing equipment with high sequence base calling enabled using MinKNOW (Version 23.04.6, Oxford Nanopore Technologies).

### 2.5 Analysis of MinION data

Basecalling and demultiplexing of POD5 files was performed by Guppy software (v 6.5.7). Low‐quality reads and <500‐ and >3000‐length reads were removed by SeqKit (v 2.5.0). Adapter trimming was performed using Porechop (v 0.2.4), and all ONT‐barcode data were eventually collected and mapped by Minimap2 (v 2.17). Single nucleotide variant calling from mapped data was obtained by Samtools (v 1.10–3) and BCFtools (v 1.5.0) via the mpileup command, and variant calling under 10× read depth was filtered. Mapped data were visualized by IGV software (v 2.16) and analyzed by Samtools and Qualimap (v 2.3). Due to the segmented influenza genome and thus comparable length of ONT reads, usage of an identical mapping process was possible. MinION data quality was documented with NanoPlot v1.41.^14^

### 2.6 Genomic dataset and phylogenetic classification

For phylogenetic tree construction of study isolates, closely related sequences of influenza A(H1N1)pdm09 and influenza A(H3N2) were downloaded from GISAID (https://gisaid.org/). For multiple sequence alignment, MAFFT software was used. For substitution model prediction, jModelTest was used. The maximum likelihood (ML) phylogenetic tree was built using IQtree (http://www.iqtree.org/).^15^ The trees were rooted with vaccine strain A/Wisconsin/588/2019 for the influenza A(H1N1)pdm09 trees and A/Darwin/6/2021 for influenza A(H3N2). For clade and subclade identification, Nextstrain was used (https://clades.nextstrain.org/).^16^ The tree was edited and visualized using the Figtree software (http://tree.bio.ed.ac.uk/software/figtree/).^17^

## 3 RESULTS

### 3.1 Total positivity rate of influenza virus from 2020 to 2023

**FIGURE 1:**
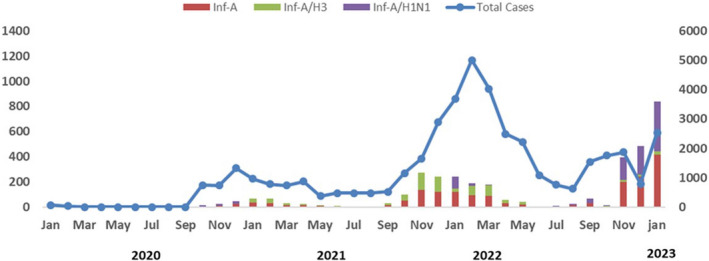
Number of influenza cases in Pakistan by month and year (January 2020–January 2023).

Between the January 1, 2020, and January 31, 2023, a total of 42,749 samples were processed, of which 1763 were influenza A positive, 1023 were A(H1N1)pdm09 positive, and 740 were influenza A(H3N2) positive. In the year 2020, the total number of samples processed was 2931, out of which 51 (1.74%) were positive for influenza A, 48 (1.63%) were positive for A(H1N1)pdm09, and 3 (0.1%) were positive for influenza A(H3N2) positive. In 2021, the total samples processed was 11,419, of which 438 (3.83%) were influenza A positive and all 438 were influenza A(H3N2) positive. In the year 2022, a total of 25,857 samples was processed, of which 855 (3.3%) were influenza A positive, 582 (2.25%) were A(H1N1)pdm09 positive, and 273 (1.05%) were influenza A(H3N2) positive. In the year 2023, in January, the total number of cases reported was 2542, of which 419 (16.5%) were positive for influenza A, 26 (1%) were positive for A(H3N2), and 393 (15.5%) were positive for A(H1N1)pdm09 (Figure 1).

Amplicons with ONT‐specific barcodes were successfully amplified by nested RT‐PCR. Finally, an average of 4,231,547 reads (total 5.3 Gb) was generated within 48 h after the start of MinION sequencing. The mean average coverage per sample for influenza A(H1N1)pdm09 virus was 928 and 897 for influenza A(H3N2) virus.

### 3.2 Phylogenetic and mutation profiling

**FIGURE 2:**
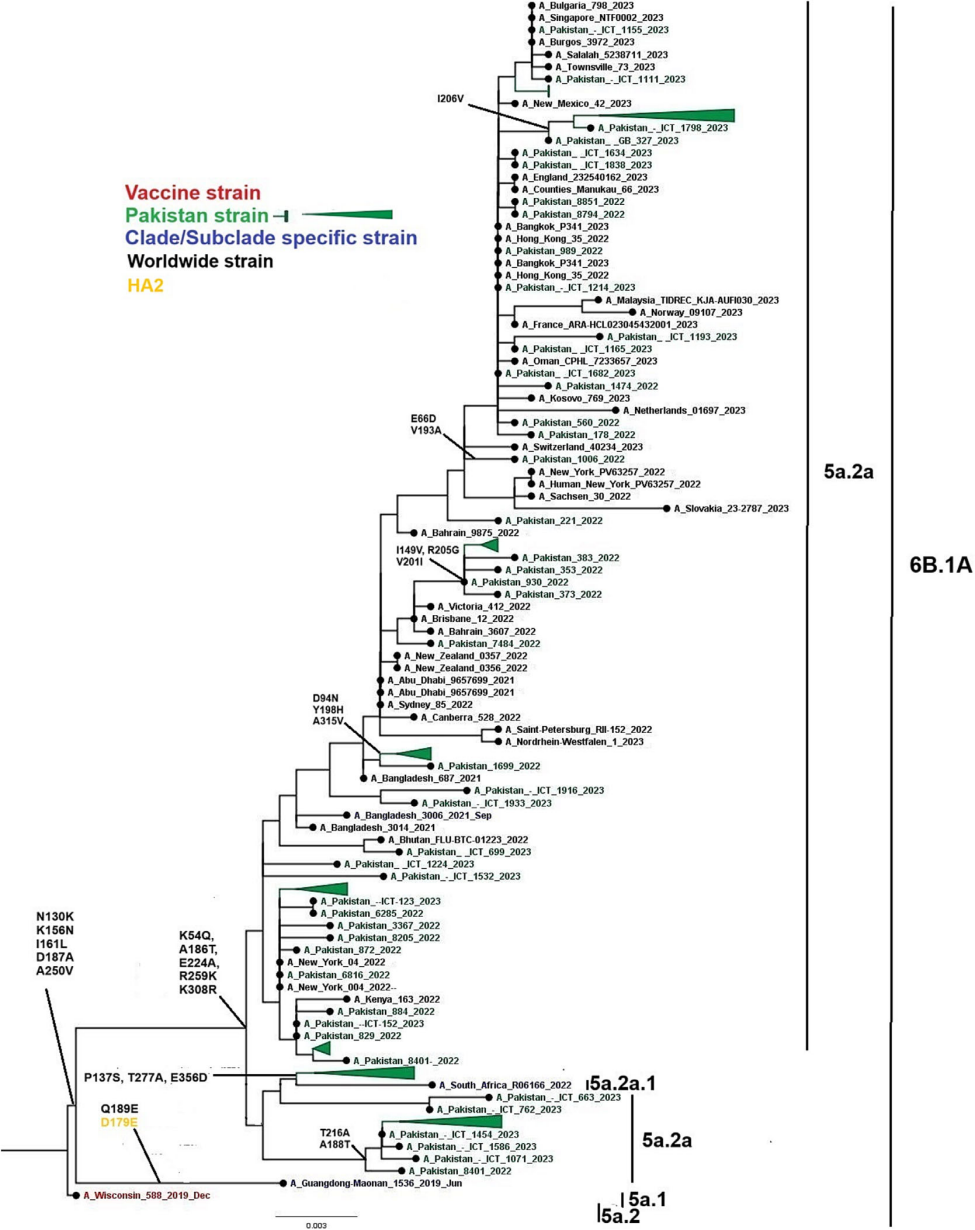
A phylogenetic tree of the Influenza A(H1N1)pdm09 HA gene. Worldwide sequences are in black, with the vaccine strain highlighted in red, study sequences in green, and subclades‐specific reference strains in blue.

Phylogenetic analysis was performed on 90 HA genes of influenza A(H1N1)pdm09 sequences obtained in this study, along with 45 worldwide isolates. The sequences from Pakistan in this study showed close similarity to sequences from Bangkok, Oman, Bangladesh, Switzerland, Bahrain, and the United States. These sequences were classified as belonging to subclade 6B.1A.5a.2a (Figure 2).

**FIGURE 3:**
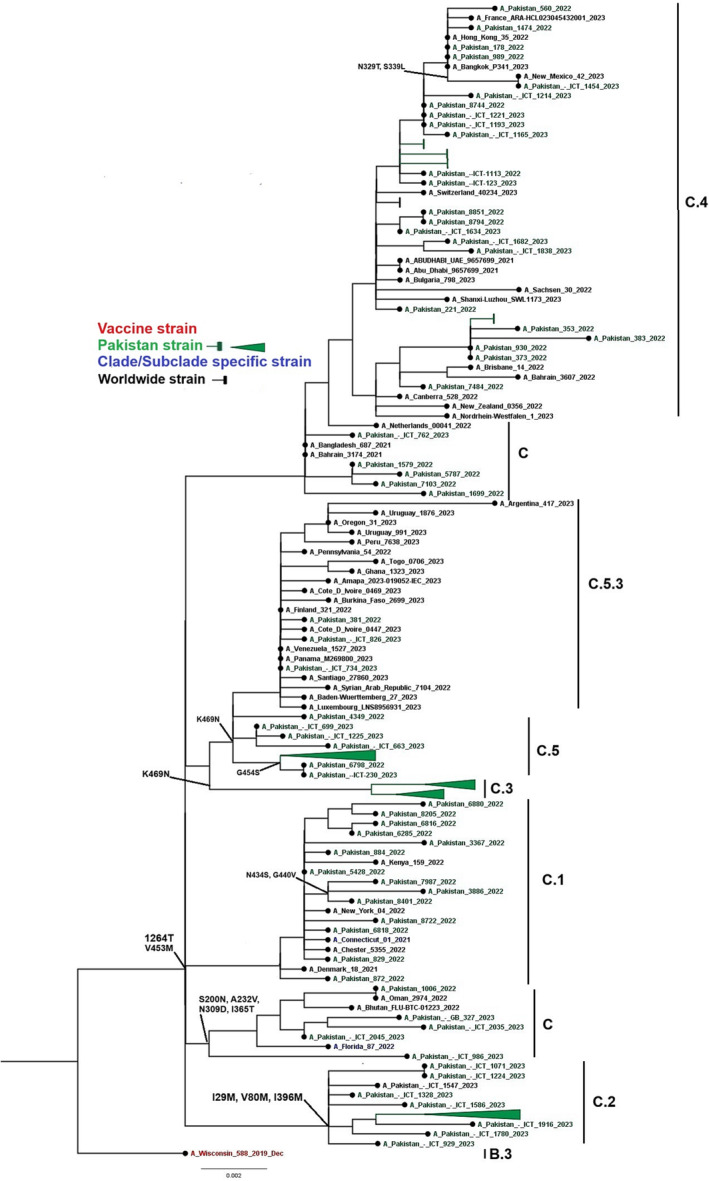
A phylogenetic tree of the Influenza A(H1N1)pdm09 NA gene. Worldwide sequences are in black, with the vaccine strain highlighted in red, study sequences in green, and subclades‐specific reference strains in blue.

A phylogenetic tree was constructed for 90 study and 45 global isolates of influenza A(H1N1)pdm09 NA gene. The study samples were classified into subclades C, C.1, C.2, C.3, C.4, C.5, and C.5.3 as defined by Nextstrain. Notably, Pakistani sequences exhibited a close genetic relationship with strains from Bangkok, Oman, Switzerland, and the United States (Figure 3). When compared to the reference vaccine strain A/Wisconsin/588/2019, the nucleotide divergence ranged from 0.0518% to 0.065%, which represents less than 1% divergence.

**FIGURE 4:**
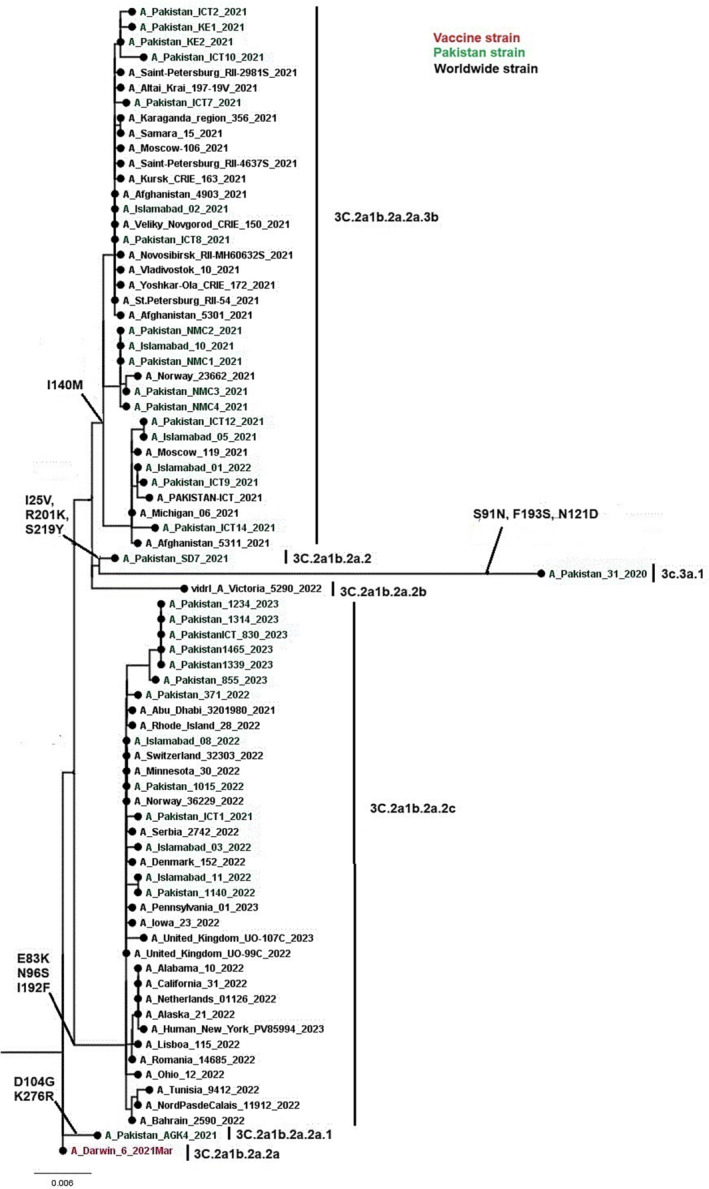
A phylogenetic tree of the Influenza A(H3N2) HA gene. Worldwide sequences are in black, with the vaccine strain highlighted in red, study sequences in green, and subclades‐specific reference strains in blue.

**FIGURE 5:**
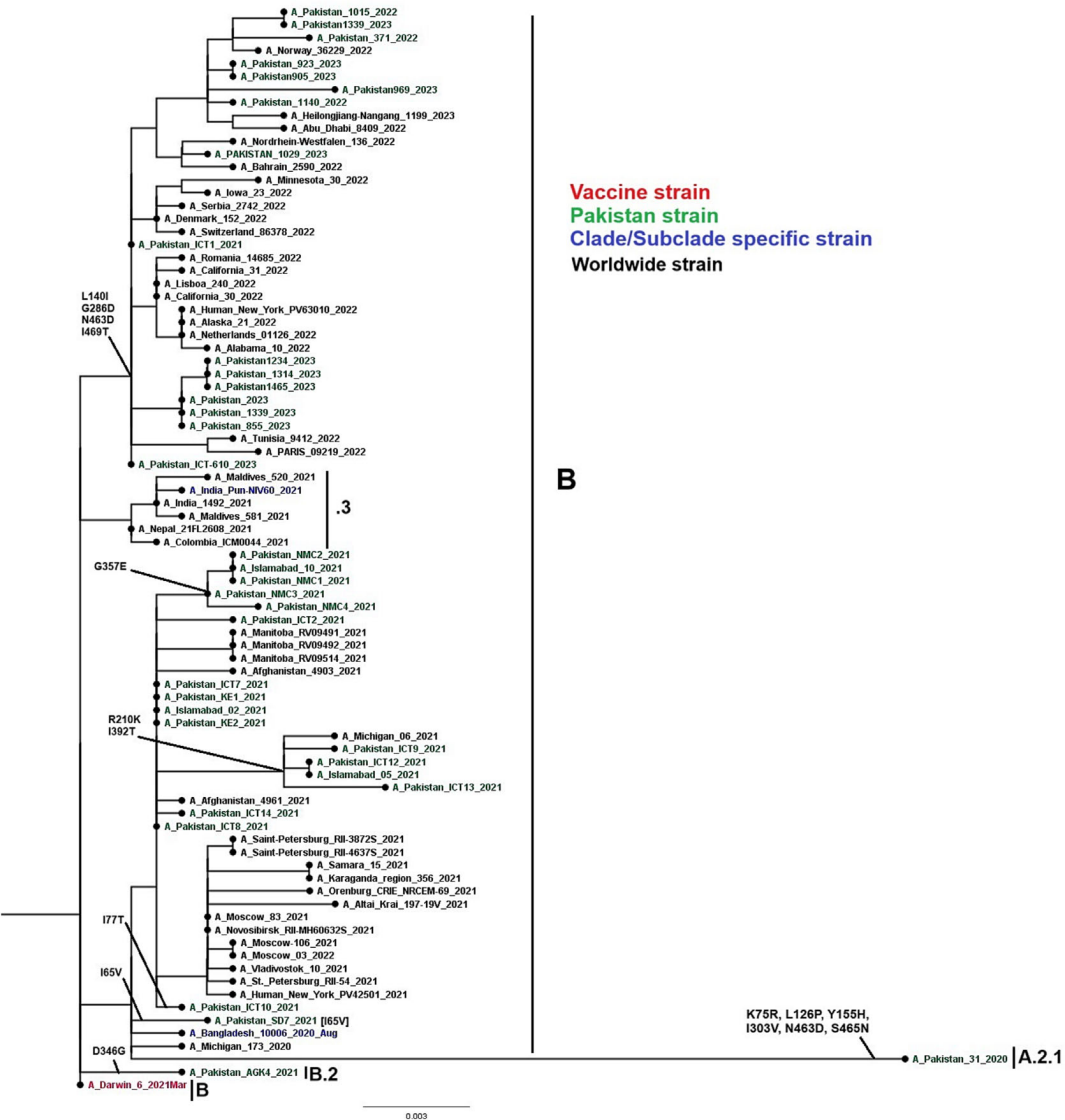
A phylogenetic analysis of the Influenza A(H3N2) NA gene. Worldwide sequences are in black, with the vaccine strain highlighted in red, study sequences in green, and subclades‐specific reference strains in blue.

Phylogenetic analysis of 76 HA genes of influenza A(H3N2) were constructed. Sequences from Pakistan were closely related to sequences of Afghanistan, Norway, and the United States (Figure 4). All study sequences of influenza A(H3N2) HA gene were from six subclades (3C.3a.1 and 3C.2a1b.2a [2, 2a.1, 2b, 2c, and 2a.3b]. A phylogenetic tree of the influenza A(H3N2) NA gene was constructed using 84 full‐length NA gene sequences of influenza A(H3N2). All study sequences of influenza A(H3N2) NA gene were from subclades A.2.1, B, and B.2 (Figure 5). Notably, Pakistani sequences clustered closely with sequences from Afghanistan, Norway, and the United States. The nucleotide divergence rate of influenza A(H3N2) from the vaccine strain A/Darwin/6/2021 ranged from 0.0249% to 0.0441%, indicating a divergence of less than 1%.

### 3.3 Amino acid variation in antigenic sites

The study sequences of 90 samples of A(H1N1)pdm09, which undergo amino acid substitutions at various positions in all eight segments of influenza, are presented in Table 2. Additionally, Table 3 displays amino acid mutations at 36 study sequenced samples of A(H3N2) in all eight segments.^16^, ^18^

**TABLE 2:** Most common amino acid mutations observed in all eight segments of A/H1N1.

MP	NP	NS	PA	HA	NA	PB1	PB2
E6Q	A22T	D2E	V63L	K54Q	V13I	G154D	R54K
V7I	V100I	E55K	V100I	A186T	N44S	V200I	M66I
E8Y	M105T	T80A	P224S	Q189E	G245X	K237R	T81I
T9R	Q398K	V84I	S225C	E224A	P246X	K386R	D195N
D21G	V425I	L90I	L226I	R259K	N386K	I397M	G225S
S23N	S498N	I111T	D294N	K308R	I389K	I435T	R293K
V80I		I123V	N321K		D416N		R299K
M192V		E125D	R362K		K432E		V344M
Q208K		K131E	I505V		N449D		I354L
K230R		A155T	G578S		T452I		S453T
E235A		N205S	R626K		V453M		V511I
							V667I
							V731I

**TABLE 3:** Most common amino acid mutations observed in all eight segments of A/H3N2.

MP	NP	NS	PA	HA	NA	PB1	PB2
V7I	I136M	R41K	L78I	G53D	E119X	K327X	M81I
G16E	V100I	S212P	N409X	E83K	Y155H	N328X	S107D
V27I	I136M	K217E	A100X	D104G	E221D	T57I	K340R
D30S	V197I	R224G	E119X	I140M	S245X	R586K	M410V
L43T	E220D	A226I	K158R	S205F	A246X	A587T	I461V
R77Q	P283X	R227G	N272S	A212T	S247X	L598X	R526K
N82S	K293X	S228T	I348L	K276R	G248X	E618D	I588T
S89G	Y313V	K229E	V354I		N329S		T613A
R101K	N373T	V230I	D396E		S367N		T711N
N133X	L418I		S402T		E368X		
T139X	S450G		N409X		N402D		
H159X	T472A		T515X		D463N		
			V668I				
			N675K				

## 4 DISCUSSION

This study represents whole‐genome sequencing of the influenza A(H1N1)pdm09 and A(H3N2) virus within Pakistan. Using WGS, we analyzed the genetic variation between vaccine and circulating strains. The phylogenies in Pakistan illustrate the antigenic drift of influenza A viruses. This research examined how viral sequence data obtained through WGS may supplement “traditional” clinical data in estimating severe influenza infection in hospitalized SARI patients.^19^, ^20^, ^21^ Furthermore, because all influenza sentinel sites were involved in the surveillance, the data are thought to be more generalizable than in single‐center studies. WGS was performed on a total of 111 (*n* = 126) samples from January 2020 to January 2023 of which 90 samples were of influenza A(H1N1)pdm09 and 36 samples were of influenza A(H3N2).

In the present study, virological data were recorded for influenza A(H1N1)pdm09. A total of 42,749 samples of influenza A was processed from 2020 to 2023: of which 2931 were processed in the year 2020, 11,419 in 2021, 25,857 in 2022, and 2542 samples in 2023. In comparison to the observed trend after the pandemic of influenza A(H1N1)pdm09 in 2009, the virus is currently co‐circulating with other influenza viruses and has relinquished its status as the primary influenza A virus in several countries.^22^, ^23^ As stated by Dapat et al.,^24^ the decline in clinical cases is linked to heightened levels of antibodies against influenza A virus within the community. In the context of Pakistan, this reduction can also be ascribed to targeted vaccination efforts of influenza A among high‐risk groups.

The NGS analysis not only provided the complete genome of the viruses but also obtained the amino acid substitutions across the eight segmented genes. Furthermore, several functional mutations of A(H1N1)pdm09 had already been identified.^18^, ^25^ Overall, our results suggest that the influenza A(H1N1)pdm09 samples studied have undergone genetic changes but are still relatively genetically close to the vaccine strain influenza A/Wisconsin/588/2019.

Mutations in the H1N1pdm09 viruses resulted in changes in the HA1 polypeptide, specifically K54Q, A186T, Q189E, E224A, R259K, and K308R. Notably, several of these substitutions occupy both antigenic and receptor binding sites, suggesting that they may have served some compensatory function that allowed the virus to improve its fitness and/or antigenic structure.^18^, ^26^, ^27^

The amino acid substitution I140M in A(H3N2) HA1 has been identified in 52% of sequenced isolates. I140M is situated within antigenic site A and has been observed to diminish the binding affinity of H3N2‐specific antibodies. Nonetheless, its precise impact on immune recognition remains to be fully elucidated.^27^, ^28^ Furthermore, among the sequenced isolates, amino acid substitutions S205F and A212T were prevalent in 67% of cases.

Pakistan sequences of this study were closely related to sequences from Bangkok, Oman, Bangladesh, Afghanistan, Bahrain, and the United States. This could be attributed to the fact that Pakistan lies in the same region of northern hemisphere as Bangkok, Oman, Bangladesh, Afghanistan, and Bahrain, and extensive travel in this region may be one of the causes.^29^

The result of the influenza A(H1N1)pdm09 phylogenetic tree with altered amino acid sequences suggests that the vaccine may be less effective against A(H1N1)pdm09 in Pakistan. Additionally, between September 13, 2022, and January 25, 2023, the US Centers for Disease Control and Prevention (CDC) revealed that the vaccination was 54% effective against laboratory‐confirmed influenza A in inpatient and emergency department (ED) settings.^30^, ^31^

In summary, NGS was effectively used to characterize the whole genomes of influenza A(H1N1)pdm09 and A(H3N2) viruses, providing high‐throughput data for phylogenetic construction, mutation analysis, and nucleotide diversity. Furthermore, numerous mutations were found in both A(H1N1)pdm09 and A(H3N2), particularly in HA genes.^18^, ^32^, ^33^

The obtained whole genome data from this study holds potential for future mutation analysis, especially through comparisons with data from other studies. Understanding factors like viral mutations could aid public health officials in predicting influenza severity early in the season, guiding safety precautions and hospital strategies. Moreover, these findings could have implications for individual patient care, allowing better identification of those at risk of severe disease for improved management and treatment, such as antiviral administration. While sentinel‐based surveillance studies may not fully represent the general population, the study's approach provides a broader perspective on similar clinical presentations, offering insights into a larger population context.

## 5 CONCLUSION

The evolutionary analysis of seasonal influenza A(H1N1)pdm09 virus highlights amino acid substitution in the vaccine strain compared to the circulating virus in Pakistan. While this study underscores the potential of compiling mutation data over multiple influenza seasons to establish a predictive mutation database, more research is needed to operationalize this approach in public health. The identified mutations offer promising avenues for further exploration using molecular biology techniques to determine their impact on virus intensity. Additionally, investigating other aspects of the viral genome, such as reassortment events and minor genetic variants within the viral RNA population, holds potential for future predictive modeling of influenza severity.

## AUTHOR CONTRIBUTIONS

*Conceived and designed the experiments*: Aamer Ikram, Muhammad Salman, and Nazish Badar. *Performed the experiments*: Nazish Badar, Hamza Ahmed Mirza, Abdul Ahad, and Sidra Saeed. *Analyzed the data*: Nazish Badar, Sidra Saeed, Abdul Ahad, and Massab Umair. *Contributed reagents/materials/analysis tools*: Aamer Ikram and Muhammad Salman. *Wrote the manuscript*: Nazish Badar, Umer Farooq, and Sidra Saeed.

## CONFLICT OF INTEREST STATEMENT

There is no conflict of interest between the author and any coauthor of this article.

### PEER REVIEW

The peer review history for this article is available at https://www.webofscience.com/api/gateway/wos/peer-review/10.1111/irv.13262.

## ETHICS STATEMENT

The Pakistan National Institute of Health Internal Review Board authorized the surveillance and sample techniques. Each subject provided written or verbal agreement; however, the patients' identities were not revealed at any point. The institutional board was appraised of the study setting's special needs for approval. The data form included a check box to document the authorization‐seeking method.

## Supporting information


**Data S1.** Supporting Information

## Data Availability

The sequences generated in the study were submitted to GISAID (126) files provided in the supporting information.
